# Comprehensive chromosome analysis of blastocysts before implantation using array CGH

**DOI:** 10.1186/1755-8166-6-22

**Published:** 2013-06-03

**Authors:** Mi Kyung Chung, Hyeon Jeong Jeong, Jung Hyun Lee, Sang-Jin Park, Hee-Doo Chung, Ho-Young Kang

**Affiliations:** 1 Seoul Rachel Fertility Center, 109 Mapo-daero, Seoul, Korea; 2 MGMED, Inc., Elysia bldg, 173, digital-ro, Gasan-dong, Seoul, Korea; 3 MGMED Clinic, 60-24 Gasan-dong, Seoul, Korea

## Abstract

**Background:**

Chromosomal abnormalities are common in embryos produced *in vitro* and cause implantation failure, miscarriage, and serious medical problems in infants. Because preimplantation genetic screening (PGS) is increasingly being used to detect aneuploidy in embryos with the purpose of improving implantation rates after IVF (*in vitro* fertilization), we aimed to validate the usefulness of array CGH for the preimplantation genetic screening (PGS) of embryos at the blastocyst stage of development.

**Results:**

A total of 150 blastocysts were biopsied from couples undergoing IVF and analyzed using array CGH. We found that 54.5% (73/134) of the blastocysts were euploid embryos, whereas 45.5% of the embryos (61/134) had chromosomal abnormalities. Multiple chromosome abnormality was most frequently observed (34.4%), and dual aneuploidy was observed in 26.2% of the embryos. Monosomy (21.3%) appeared more frequently than trisomy (18%).

**Conclusion:**

Chromosomal microarray analysis provided clinically significant cytogenetic information regarding the frequency and variety of chromosomal abnormalities observed in embryos at the blastocyst stage, suggesting that this is a useful tool for comprehensive aneuploidy screening in IVF.

## Background

During IVF procedures, a preimplantation genetic diagnosis (PGD) is used to eliminate embryos carrying genetic diseases prior to implantation. The first application of PGD was successfully performed for couples at risk for transmitting recessive X-linked diseases to male offspring [1], whereby polymerase chain reaction (PCR) was used to determine the sex of the embryos.

Unlike PCR methods, preimplantation genetic screening (PGS) aims to provide a means for identifying potentially viable euploid embryos i.e., screening that may improve pregnancy rates. PGS was first described by Verlinsky *et al*. [2] and Munne *et al*. [3]. Although previous methods for embryo screening used fluorescence *in situ* hybridization (FISH) to analyze chromosomes [4,5], the FISH approach is limited because the technique is unable to screen all chromosomes simultaneously. Conventional comparative genomic hybridization (CGH) has been used to comprehensively screen for aneuploidy in oocytes and embryos [6,7]. However, although useful for selecting euploid embryos, the CGH protocol is not generally used because it is time consuming and complicated. At present, both array CGH (aCGH) and single nucleotide polymorphism (SNP) arrays have been validated as accurate methods for producing comprehensive analyses of chromosome in embryos that are compatible with day-3 biopsies and day-5 replacements in a fresh cycle [8-12]. The difference in mosaicism between embryos at days 3 and 5 has led to a preference for biopsies at the blastocyst stage [13,14].

Here, we describe the results of an embryo analysis and the details of the chromosomal abnormalities found.

## Results

In total, we analyzed 150 blastocysts from 49 couples undergoing IVF (Table 1). Amplification was not detected in 11 (7.3%) embryos, and noisy profile results were obtained for 3.6% (5/139) of the embryos. Euploidy was found in 54.5% of the embryos (73/134), whereas chromosomal abnormalities were found in 45.5% (61/134) of the embryos.

**Table 1:** Array-CGH results

	**Number**
Embryos analyzed	150
Euploid embryos	73
Aneuploid embryos	61
Embryos without amplification	11
Embryos with noisy profile	5

**Figure 1:**
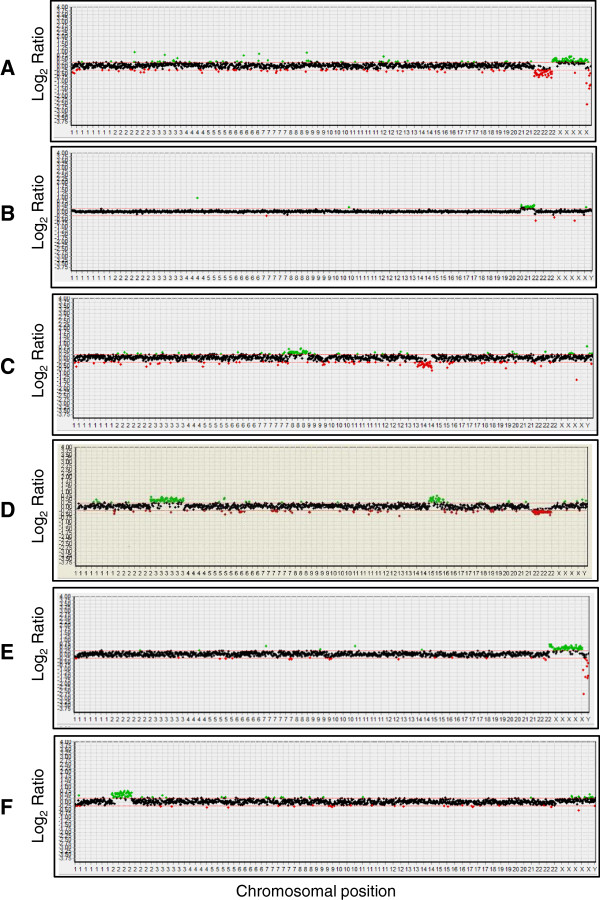
**Examples of array CGH results are shown.** (**A**) Array result displaying a female cell with the loss of chromosome 22. (**B**) A male cell with trisomy 21. (**C**) An XY cell showing two chromosomal abnormalities i.e., gain of chromosome 8 and loss of chromosome 14. (**D**) A male cell with multiple chromosomal abnormalities - gain of chromosomes 3 and 15 and loss of chromosome 22. (**E**) A normal female cell. (**F**) A cultured cell with a 2p duplication was used as a positive control.

The details of the array CGH results derived from aneuploid embryos (n=61) are summarized in Table 2. The type of chromosomal abnormality that was most frequently observed was multiple chromosomal abnormality (34.4%), and the second most frequent was dual chromosomal abnormality (26.2%). Monosomy (21.3%) appeared more frequently than trisomy (18%). Examples of array CGH profiles are shown in Figure 1.

**Table 2:** Chromosome abnormality analyzed

**Aneuploid types**	**Number (%)**
Single chromosome loss	13 (21.3%)
Single chromosome gain	11 (18.0%)
Dual chromosomal abnormality	16 (26.2%)
Multiple chromosomal abnormality	21 (34.4%)

The chromosomes that were most frequently detected to have aneuploidy were, in order, 15, 22, 21, 16, and 18. Chromosomes 4 and 12 were the least frequently found to have aneuploidy.

## Discussion

Preimplantation genetic screening for aneuploidy is increasingly used to examine the chromosomes of embryos from couples undergoing IVF [8-10,15,16]. The purpose of PGS is to identify embryos that are free from chromosomal abnormalities. The main indications for PGS are maternal age, repeated implantation failure, and repeated miscarriage. We examined 150 embryos from 49 couples with these indications.

As shown in Table 1, we successfully analyzed 89.3% (134/150) of the embryos and found that 45.5% (61/134) of the embryos contained abnormal chromosomes. Although the array CGH method is robust and specific, we observed some failure in amplification and a noisy profile. Some cells containing degraded DNA or samples of low quality resulting from apoptosis can be obtained during the biopsy procedure, causing experimental error.

The results showed excessive single chromosome loss *versus* single chromosome gain (Table 2); frequent abnormalities in chromosomes 15, 22, 21, 16, and 18; and rare aneuploidy in chromosomes 4 and 12, which are similar to the results of previous reports [17-19]. Although the frequency of chromosomal abnormalities varied, aneuploidy occurred in all of the 24 chromosomes (data not shown), suggesting that PGS is necessary for selecting healthy embryos during IVF procedures. Previous studies have shown the importance of screening embryos with improved pregnancy success as a result [19].

In conclusion, array CGH is a useful technique for the detection of chromosomal abnormalities during IVF procedures, as previously described [18]. However, embryo cultures up to days 5 or 6 should be established before performing array CGH experiments on blastocysts, and further evidence is required to determine whether PGS results in enhanced delivery rates[14].

## Methods

### Patient materials

A total of 150 blastocysts were collected from 49 couples who visited the clinic center to undergo IVF between September 2011 and December 2012. All patient materials were obtained and evaluated with informed patient consent and under approval from the Ethics Committees of MGMED clinic center and Seoul Rachel Fertility Center. All patients were provided with counseling regarding PGS using array CGH and signed an informed consent prior to entering the study.

### Experimental procedures

The biopsied cells were washed in PBS and collected into PCR tubes. Whole-genome amplification was performed using a kit and following the manufacturer’s instructions (Sigma-Aldrich, Saint Louis, MO).

Approximately 3 μg of amplified DNA was used in the array CGH experiments, as described, with slight modifications [20]. Briefly, the amplified DNA was labeled with Cy-3 and Cy-5 dCTP for 3 h using a random priming method. The labeled DNA was purified, dissolved in hybridization buffer, and hybridized overnight. The slides were washed several times and dried as described [21]. Images of the slides were acquired with a GenePix4000B dual-laser scanner (Axon Instruments, Union City, CA) and analyzed with MacViewer software [21].

## Competing interest

The authors declare that they have no competing interests.

## Authors’ contributions

MKC and SJP analyzed the data for the paper. HDJ and HJJ helped with the discussion and data summary. JHL performed various experiments. HYK drafted the manuscript, conceived of the study, and also approved the manuscript. All authors read and approved the final manuscript.

## References

[B1] Handyside AH Kontogianni EH Hardy K Winston RM Pregnancies from biopsied human preimplantation embryos sexed by Y-specific DNA amplification Nature 1990 344 768 770 10.1038/344768a0 2330030

[B2] Verlinsky Y Cieslak J Freidine M Ivakhnenko V Wolf G Kovalinskaya L White M Lifchez A Kaplan B Moise J Pregnancies following pre-conception diagnosis of common aneuploidies by fluorescent in-situ hybridization Hum Reprod 1995 10 1923 1927 8583011 10.1093/oxfordjournals.humrep.a136207

[B3] Munne S Dailey T Sultan KM Grifo J Cohen J The use of first polar bodies for preimplantation diagnosis of aneuploidy Hum Reprod 1995 10 1014 1020 7650111 10.1093/oxfordjournals.humrep.a136027

[B4] Colls P Goodall N Zheng X Munne S Increased efficiency of preimplantation genetic diagnosis for aneuploidy by testing 12 chromosomes Reprod Biomed Online 2009 19 532 538 10.1016/j.rbmo.2009.05.002 19909595

[B5] Jansen RP Bowman MC de Boer KA Leigh DA Lieberman DB McArthur SJ What next for preimplantation genetic screening (PGS)? Experience with blastocyst biopsy and testing for aneuploidy Hum Reprod 2008 23 1476 1478 10.1093/humrep/den129 18539624

[B6] Voullaire L Wilton L Slater H Williamson R Detection of aneuploidy in single cells using comparative genomic hybridization Prenat Diagn 1999 19 846 851 10.1002/(SICI)1097-0223(199909)19:9<846::AID-PD657>3.0.CO;2-# 10521843

[B7] Wells D Delhanty JD Comprehensive chromosomal analysis of human preimplantation embryos using whole genome amplification and single cell comparative genomic hybridization Mol Hum Reprod 2000 6 1055 1062 10.1093/molehr/6.11.1055 11044470

[B8] Hellani A Abu-Amero K Azouri J El-Akoum S Successful pregnancies after application of array-comparative genomic hybridization in PGS-aneuploidy screening Reprod Biomed Online 2008 17 841 847 10.1016/S1472-6483(10)60413-0 19079969

[B9] Hu DG Webb G Hussey N Aneuploidy detection in single cells using DNA array-based comparative genomic hybridization Mol Hum Reprod 2004 10 283 289 10.1093/humrep/gah038 14985479

[B10] Le Caignec C Spits C Sermon K De Rycke M Thienpont B Debrock S Staessen C Moreau Y Fryns JP Van Steirteghem A Single-cell chromosomal imbalances detection by array CGH Nucleic Acids Res 2006 34 e68 10.1093/nar/gkl336 16698960 PMC3303179

[B11] Johnson DS Gemelos G Baner J Ryan A Cinnioglu C Banjevic M Ross R Alper M Barrett B Frederick J Preclinical validation of a microarray method for full molecular karyotyping of blastomeres in a 24-h protocol Hum Reprod 2010 25 1066 1075 10.1093/humrep/dep452 20100701 PMC2839907

[B12] Treff NR Su J Tao X Miller KA Levy B Scott RT Jr A novel single-cell DNA fingerprinting method successfully distinguishes sibling human embryos Fertil Steril 2010 94 477 484 10.1016/j.fertnstert.2009.03.067 19394599

[B13] Schoolcraft WB Fragouli E Stevens J Munne S Katz-Jaffe MG Wells D Clinical application of comprehensive chromosomal screening at the blastocyst stage Fertil Steril 2010 94 1700 1706 10.1016/j.fertnstert.2009.10.015 19939370

[B14] Ly KD Agarwal A Nagy ZP Preimplantation genetic screening: does it help or hinder IVF treatment and what is the role of the embryo? J Assist Reprod Genet 2011 28 833 849 10.1007/s10815-011-9608-7 21743973 PMC3169679

[B15] Hodes-Wertz B Grifo J Ghadir S Kaplan B Laskin CA Glassner M Munne S Idiopathic recurrent miscarriage is caused mostly by aneuploid embryos Fertil Steril 2012 98 675 680 10.1016/j.fertnstert.2012.05.025 22683012

[B16] Ata B Kaplan B Danzer H Glassner M Opsahl M Tan SL Munne S Array CGH analysis shows that aneuploidy is not related to the number of embryos generated Reprod Biomed Online 2012 24 614 620 10.1016/j.rbmo.2012.02.009 22503277

[B17] Munne S Bahce M Sandalinas M Escudero T Marquez C Velilla E Colls P Oter M Alikani M Cohen J Differences in chromosome susceptibility to aneuploidy and survival to first trimester Reprod Biomed Online 2004 8 81 90 10.1016/S1472-6483(10)60501-9 14759293

[B18] Gutierrez-Mateo C Colls P Sanchez-Garcia J Escudero T Prates R Ketterson K Wells D Munne S Validation of microarray comparative genomic hybridization for comprehensive chromosome analysis of embryos Fertil Steril 2011 95 953 958 10.1016/j.fertnstert.2010.09.010 20971462

[B19] Yang Z Liu J Collins GS Salem SA Liu X Lyle SS Peck AC Sills ES Salem RD Selection of single blastocysts for fresh transfer via standard morphology assessment alone and with array CGH for good prognosis IVF patients: results from a randomized pilot study Mol Cytogenet 2012 5 24 10.1186/1755-8166-5-24 22551456 PMC3403960

[B20] Park SJ Jung EH Ryu RS Kang HW Ko JM Kim HJ Cheon CK Hwang SH Kang HY Clinical implementation of whole-genome array CGH as a first-tier test in 5080 pre and postnatal cases Mol Cytogenet 2011 4 12 10.1186/1755-8166-4-12 21549014 PMC3114015

[B21] Choe J Kang JK Bae CJ Lee DS Hwang D Kim KC Park WY Lee JH Seo JS Identification of origin of unknown derivative chromosomes by array-based comparative genomic hybridization using pre- and postnatal clinical samples J Hum Genet 2007 52 934 942 10.1007/s10038-007-0199-1 17940726

